# The lipid peroxidation derived DNA adduct γ-OHPdG levels in paraneoplastic liver tissues predict postoperative outcomes of hepatoma

**DOI:** 10.7150/jca.56982

**Published:** 2021-05-13

**Authors:** Yang-Yi Feng, Jen Yu, Yi Hsuan Huang, Yang-Hsiang Lin, Chau-Ting Yeh

**Affiliations:** 1 Department of Gastroenterology & Hepatology, Chang Gung Memorial Hospital, Linkou, Taoyuan, Taiwan.; 2 Department of internal medicine, Chang Gung Memorial Hospital, Linkou, Taoyuan, Taiwan.; 3 College of Medicine, Chang Gung University, Taoyuan, Taiwan.; 4 Liver Research Center, Chang Gung Memorial Hospital, Linkou, Taoyuan, Taiwan.

**Keywords:** hepatocellular carcinoma, lipid peroxidation, γ-OHPdG, survival outcome, biomarker

## Abstract

Chronic necroinflammation in liver induces lipid peroxidation and oxidative stress, which contributes to hepatocellular carcinoma (HCC) development. Gamma-hydroxy-1, N2-propanodeoxyguanosine (γ-OHPdG), a promutagenic DNA adduct, is derived from lipid peroxidation. Little is known about the clinical roles of γ-OHPdG in relationship to HCC progression. Here, we showed that γ-OHPdG levels were highly expressed in the cancerous HCC tissues (*P* = 0.020, compared to those in noncancerous parts). Postoperative outcome analysis revealed that higher γ-OHPdG expression in the paraneoplastic noncancerous tissues was independently associated with shorter distant metastasis-free survival (*P* = 0.020). In subgroup analysis, higher γ-OHPdG expression in the noncancerous tissues in hepatitis B related HCC subgroup was associated with shorter overall survival (*P* = 0.016) and distant metastasis-free survival (*P* = 0.006). However, in patient subgroups including non-cirrhosis, bilirubin < 1.2 mg/dL, alanine transaminase < 41 U/L, or aspartate transaminase < 31 U/L, higher γ-OHPdG expression in the cancerous tissues was associated with longer overall survival (P < 0.03 for all). *In vitro* experiments showed that cell viability was suppressed upon hydrogen peroxide treatment in liver cancer cell lines. In conclusion, lipid peroxidation derived marker, γ-OHPdG, in the paraneoplastic noncancerous and cancerous liver tissues predicted postoperative outcomes in HCC patients.

## Introduction

Hepatocellular carcinoma (HCC) ranks the third most frequent cause of cancer-related mortality in the world. In Taiwan, viral hepatitis accounts for more than 90% of HCC etiologies. About 3.50 million people are hepatitis B virus (HBV) carriers and 1.70 million people have hepatitis C virus (HCV) infections in Taiwan 1. To date, neonatal vaccination, nucleoside analogue therapy, direct acting antiviral and pegylated interferon treatment together have significantly reduce the prevalence of viral-related HCC 2, 3. Metabolic syndromes, including obesity, dyslipidemia and diabetes, have been correlated with an increased risk of liver diseases and HCC 4. All of them can trigger chronic necroinflammation, a pivotal factor for development of HCCs 5.

Chronic liver necroinflammation can lead to fibrosis, cirrhosis and eventually HCC. It is well-known that chronic inflammation induces oxidative stress and lipid peroxidation (LPO), producing reactive oxygen species (ROS), and subsequently enhances promutagenic DNA adducts formation, as an early important step in oncogenesis 6, 7. Gamma-hydroxy-1, N2-propanodeoxyguanosine (γ-OHPdG), a promutagenic DNA adduct, produced by LPO serves as an endogenous DNA damage indicator 8. γ-OHPdG accumulation leads to induction of G to T and G to A mutations. Feng and co-workers demonstrated that γ-OHPdG formation mainly occurred at tumor suppressor gene *TP53* that coincide with its mutation hotspots in lung cancer of smokers 9, 10. Another study indicated that antioxidants treatment reduced γ-OHPdG levels and prevented liver cancer formation in a nucleotide excision repair (NER)-deficient mouse model. In this model, they also found a higher frequency of GC > TA mutations, determined by whole exome sequencing 11. Accordingly, understanding the links between promutagenic DNA adduct, γ-OHPdG, and cancer progression is an important issue in cancer biology.

Recently, many advanced therapeutics, including target therapy and immunotherapy have been developed 12. These therapies combining with the other traditional treatments such as radiofrequency ablation and transcatheter arterial chemoembolization may improve the outcomes of patients with HCC. DNA damage response has been proposed to be a crucial event in oncogenesis and promising strategies against tumor formation can be developed 13. However, few studies, particularly in liver cancer, have been conducted regarding its clinical relevance. Our objective in our study was to explore the association between the DNA damage indicator, γ-OHPdG, and postoperative outcomes in a retrospective cohort of HCCs.

## Materials and Methods

### Patient samples

Formalin-fixed paraffin embedded (FFPE) liver specimens were obtained from 232 patients having received surgical removal of HCCs at Chang Gung Memorial Hospital. In the current study, the inclusion criteria for clinical observation were as followed: complete clinicopathological parameters, diagnosis of HCC, accurate medical records and regular follow‑up. The exclusion criteria for clinical observation were as followed: coexisting with other malignancies prior to HCC resection, questionable diagnosis of HCC, and pregnant patient. The tissue samples were retrieved from tissue bank under approval of IRB (201900261B0). Histological findings in all samples of this study were recorded based on evaluations (hematoxylin and eosin staining) by qualified clinical pathologists. Clinical parameters of the patients enrolled were shown in Table 1, including age, gender, cirrhosis, alpha-fetoprotein (AFP), viral status (hepatitis B virus [HBV] infection was diagnosed by positive HBV surface antigen; hepatitis C virus [HCV] infection was diagnosed by positive anti-HCV antibodies; non-B, non-C (NBNC) was diagnosed by negative for either marker; and HBV+HCV was recognized by the presence of both markers), bilirubin, alanine transaminase (ALT), aspartate transaminase (AST), prothrombin time, alcoholism, tumor number, the largest tumor size, Edmondson histology grade, microvascular invasion, and macrovascular invasion.

### Immunohistochemistry (IHC) staining

IHC was performed by using anti-γ-OHPdG antibody (a gift from Dr Fung-Lung Chung, Department of Oncology, Lambardi Comprehensive Cancer Center, Georgetown University, Washington, DC) as described previously 14. In brief, slides were incubated with 3% hydrogen peroxide for 15 min and pre-incubated with normal serum in phosphate-buffered saline (PBS) for 40 min. Subsequently, it was incubated with the γ-OHPdG antibodies for 1 h at room temperature. After washed with PBS for three times, 5 min each, the sections were incubated in PBS with the secondary antibodies at room temperature for 40 min. Visualization was achieved by using VECTASTAIN Elite ABC HRP kit. Positive γ-OHPdG signals were presented as brown-colored nuclei. The histology and scoring procedure were performed as described previously 15. In brief, the staining intensity was analyzed and evaluated by a computer program (Image J, NIH, Maryland, USA). The nuclear intensity of γ-OHPdG on noncancerous tissues and the cancerous tissues was graded as absent (0), weak (1+), medium (2+) or intense (3+). The percentage of positive cells, depending on γ-OHPdG stained nuclei, were graded as absent (0), focal (≤10%), regional (11%-50%) or diffuse (> 50%). The histoscore was analyzed by multiplying the percentage of γ-OHPdG-positive cells by the intensity. The median histoscore of each sample was used as the cut‐off value to divide the low/high expression groups for further clinical analysis.

### Cell viability assays

J7 and Hep3B cells (2×10^3^ /well) were grown on 96-well plates. After treatment with H_2_O_2_, cell viability was measured using 3-(4,5-dimethylthiazol-2-yl)-2,5-diphenyltetrazolium bromide (MTT) assay (Promega, Madison, WI). Absorbance was detected at 570 nm with the Titertek Multiskan Plus MK1-ELISA reader (Labsystems and Life Science International Ltd, Haver Hill, UK). Background signal was detected at 650 nm. The J7 and Hep3B parental cell lines in absence of H_2_O_2_ treatment were used as control groups.

### *In vitro* migration assay

Cell migration assay was performed by using the *in vitro* Transwell assay (Becton-Dickinson, Franklin Lakes, NJ, USA) with 8 µm pore membrane insert. Cell number was adjusted to 5 ×10^5^ cells/ml in 100 μl suspension with serum-free DMEM, and the cells were seeded on upper chambers of the Transwell plate (Becton-Dickinson). The medium in the lower chamber was DMEM supplemented with 20% FBS. The cells were incubated for 16-20 h at 37 °C and allowed to migrate to the lower chamber. Migratory cells were detected via crystal violet staining and counted using Image J software.

### Immunoblot analysis

The immunoblot procedure was assayed as described previously 16. Antibodies specific for cleaved PARP-1 (BD Biosciences, San Jose, CA, USA), and GAPDH (Merck Millipore, Billerica, MA, USA) were used. Signal intensities were analyzed using Image Gauge software (Fujifilm, Tokyo, Japan). The intensities of target genes were normalized to GAPDH signals.

### Statistical analysis

Statistical analysis was performed by SPSS version 25 (SPSS Inc., Chicago, IL, USA). Survival outcomes were evaluated based on Kaplan-Meier survival analysis. Kaplan-Meier survival curves for overall survival (OS), intrahepatic recurrence-free survival (RFS) and distant metastasis-free survival were compared using the log-rank test. *P* values < 0.05 were considered significant. Univariate and multivariate Cox's proportional hazard models were used to calculate the hazard ratio (HR) and confidence interval (CI) in survival analysis, for all patients and for subgroups.

## Results

### γ-OHPdG is an independent prognostic factor for distant metastasis-free survival

**Figure 1:**
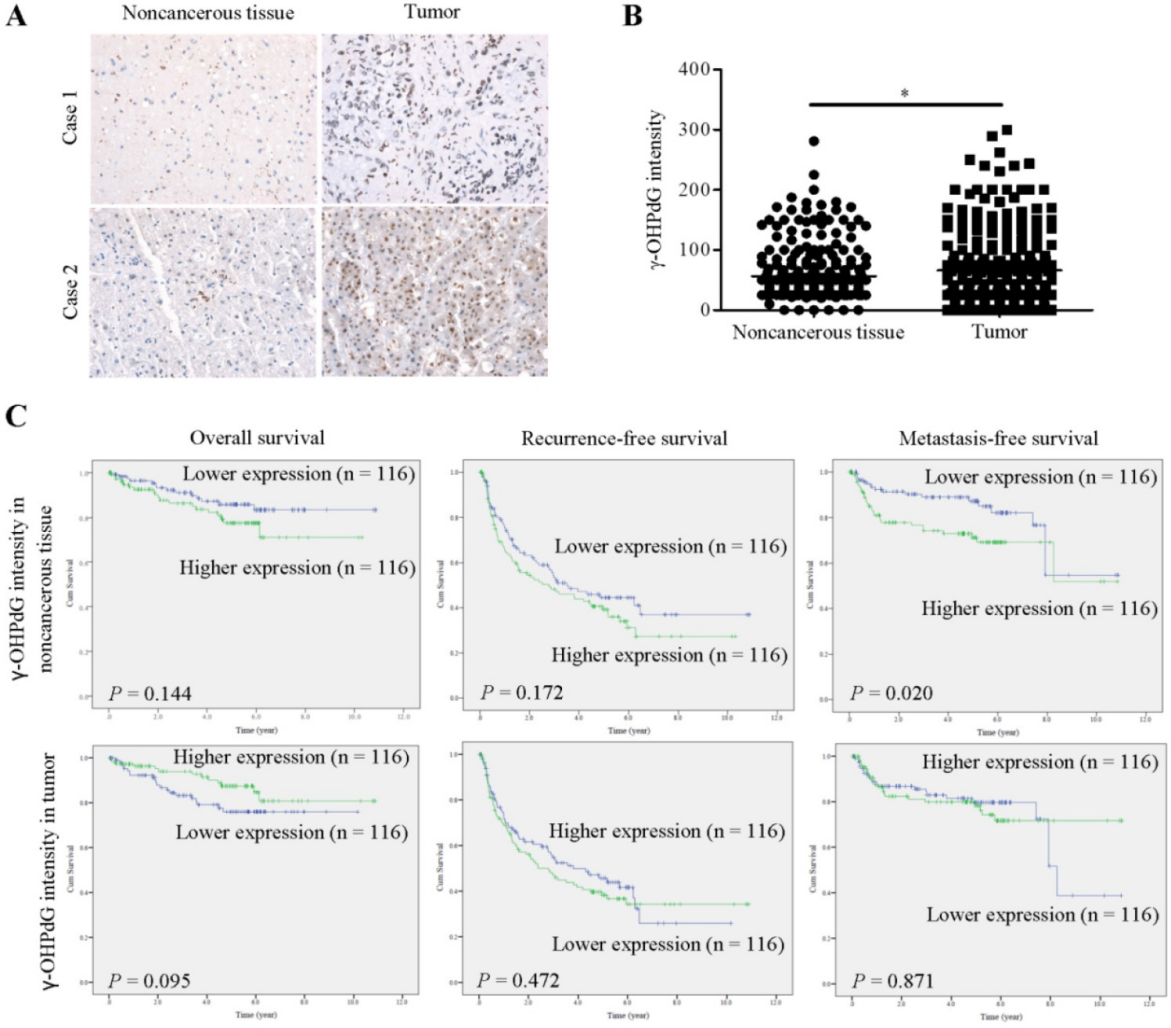
γ-OHPdG is correlated with distant metastasis-free survival in patient with HCC. (A) Expression of γ-OHPdG in paraneoplastic noncancerous tissues and cancerous tissues were assayed via IHC staining. (B) Computer-assisted quantification results of γ-OHPdG expression levels (histoscore, see Materials and Methods) were compared between noncancerous and cancerous tissues. *p < 0.01. (C) Kaplan-Meier analysis of postoperative outcomes based on γ-OHPdG expression in noncancerous and cancerous tissues. Median values were used as cutoffs. Survival outcomes were calculated by log-rank test.

To explore whether γ-OHPdG was associated HCC progression, the IHC signals in HCC tissues and their association with postoperative outcomes were analyzed. The 232-paired FFPE liver tissue samples, described in Table 1, were assessed for γ-OHPdG expression using IHC staining and scored by histological evaluation as described in Materials and Methods section. The signals of γ-OHPdG in the paraneoplastic adjacent noncancerous tissues and the cancerous tissues were detected in the nuclei as shown in Figure 1A. γ-OHPdG expression was significantly increased in the cancerous tissues compared to that in the adjacent noncancerous tissues (P = 0.020) (Figure 1B). Kaplan-Meier plot with log-rank analysis showed that higher expression of γ-OHPdG (median value as the cutoff) in the noncancerous tissues was associated with shorter distant metastasis-free survival (higher versus lower γ-OHPdG expressors, P = 0.020), but not associated with overall and intrahepatic recurrence-free survival (Figure 1C). Surprisingly, there were no statistically significant associations between γ-OHPdG expression in the cancerous HCC tissues and all three survival outcomes (Figure 1C, lower panels). Based on this observation, patients were divided into γ-OHPdG higher and lower expressors, in the noncancerous and cancerous tissues respectively, for subsequent analysis (Table 2). Univariate Cox's proportional hazard models indicated that γ-OHPdG levels in noncancerous tissues (P = 0.023), tumor number (P = 0.039), microvascular invasion (P < 0.001) and macrovascular invasion (P = 0.007) were positively correlated with distant metastasis-free survival. Furthermore, only γ-OHPdG levels in noncancerous tissues, microvascular invasion and macrovascular invasion status served as independent prognostic factors associated with distant metastasis-free survival (adjusted P = 0.030, 0.001, and 0.033, respectively) (Table 2). Our findings suggested that the γ-OHPdG expression in pre-cancerous stage might serve as an indicator for postoperative metastasis in HCC progression.

### Expression of γ-OHPdG in noncancerous tissues is an effective prognosis predictor in subgroups of HCC

**Figure 2:**
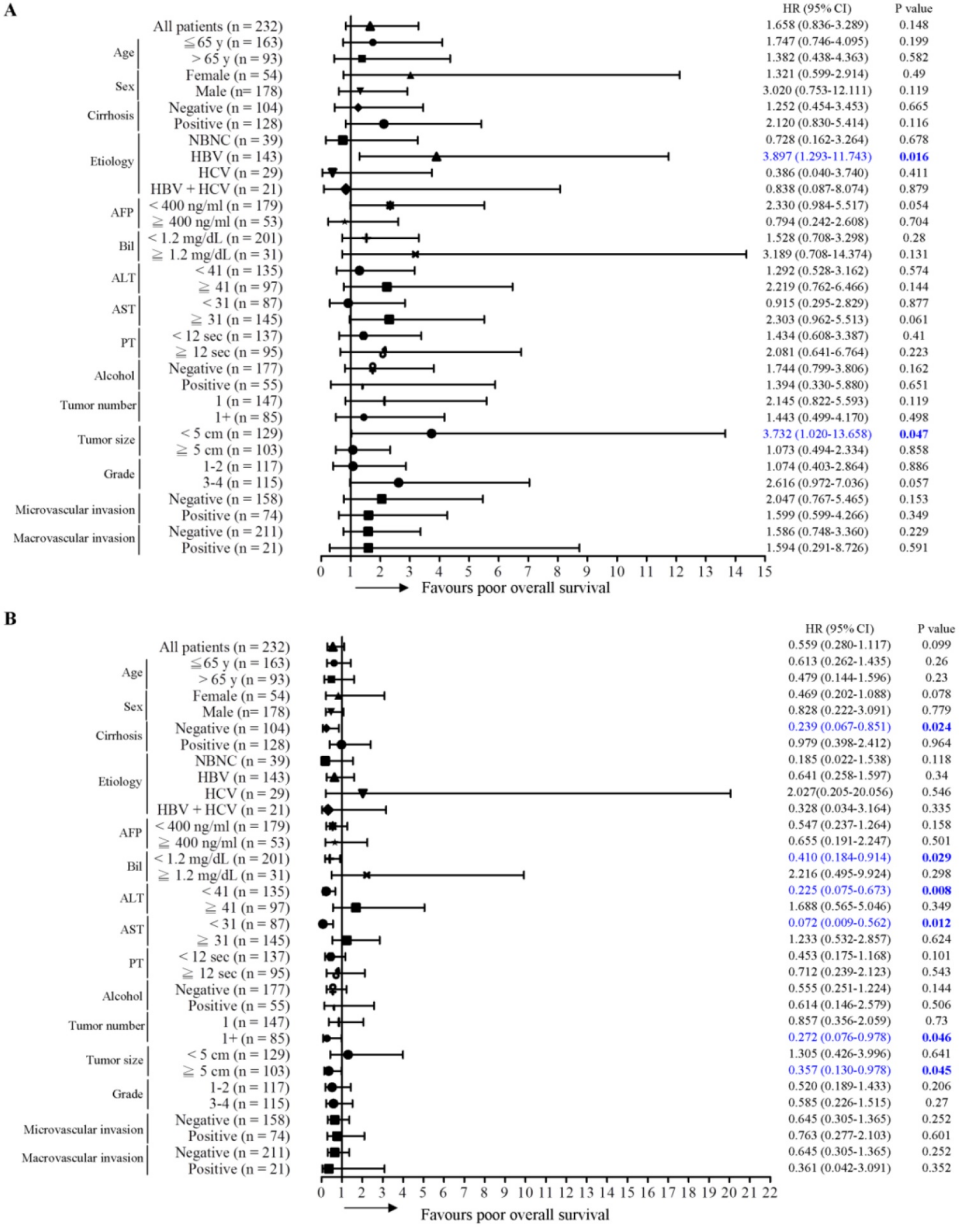
Association between γ-OHPdG expression and overall survival was analyzed by forest plot analysis in various clinical subgroups. The model of Cox proportional hazard method was used to analyze γ-OHPdG levels in (A) noncancerous tissues and (B) cancerous tissues in relationship to overall survival. Horizontal lines represent 95% confidence intervals.

**Figure 3:**
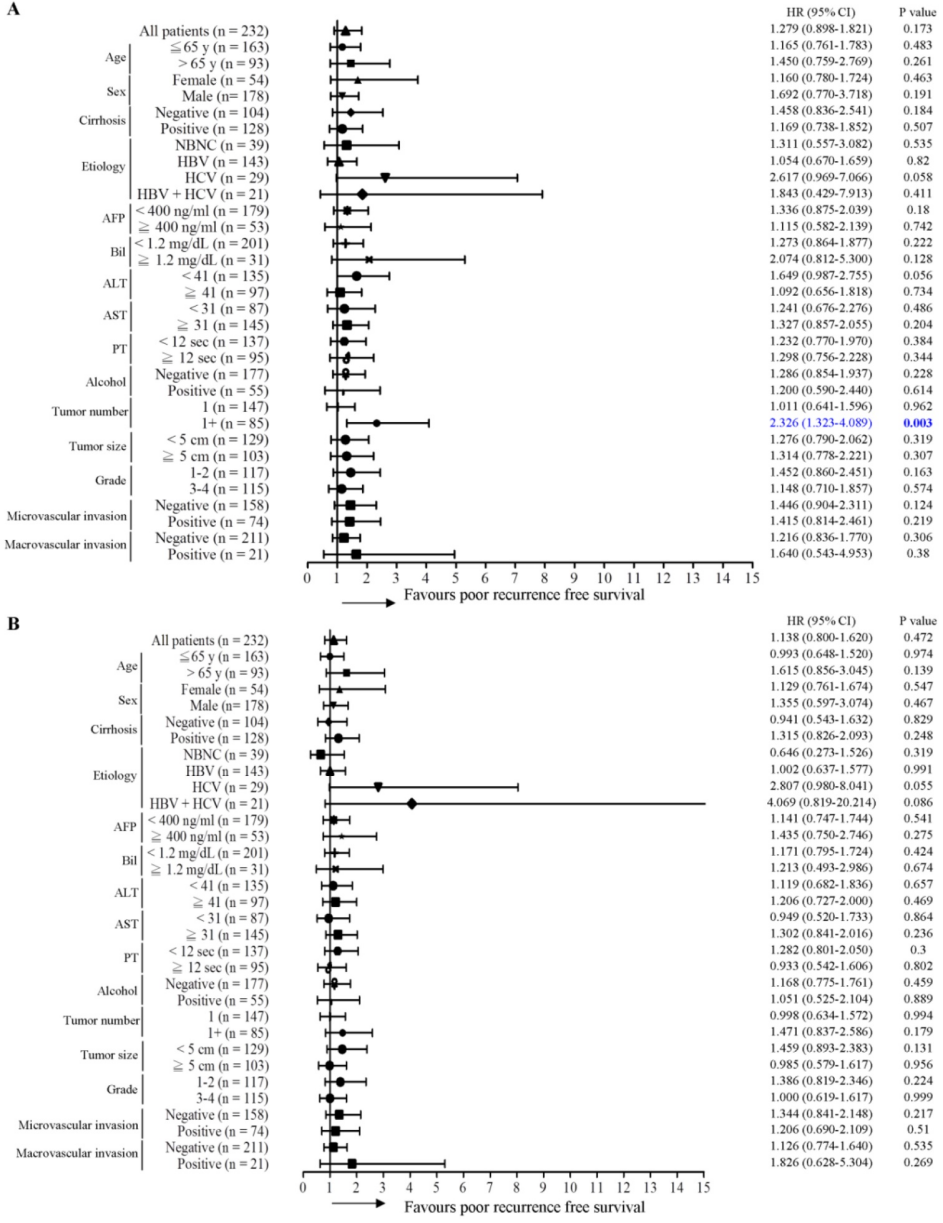
Association between γ-OHPdG expression and intrahepatic recurrence-free survival was analyzed by forest plot analysis in various clinical subgroups. The model of Cox proportional hazard method was used to analyze γ-OHPdG levels in (A) noncancerous tissues and (B) cancerous tissues in relationship to intrahepatic recurrence-free survival. Horizontal lines represent 95% confidence intervals.

**Figure 4:**
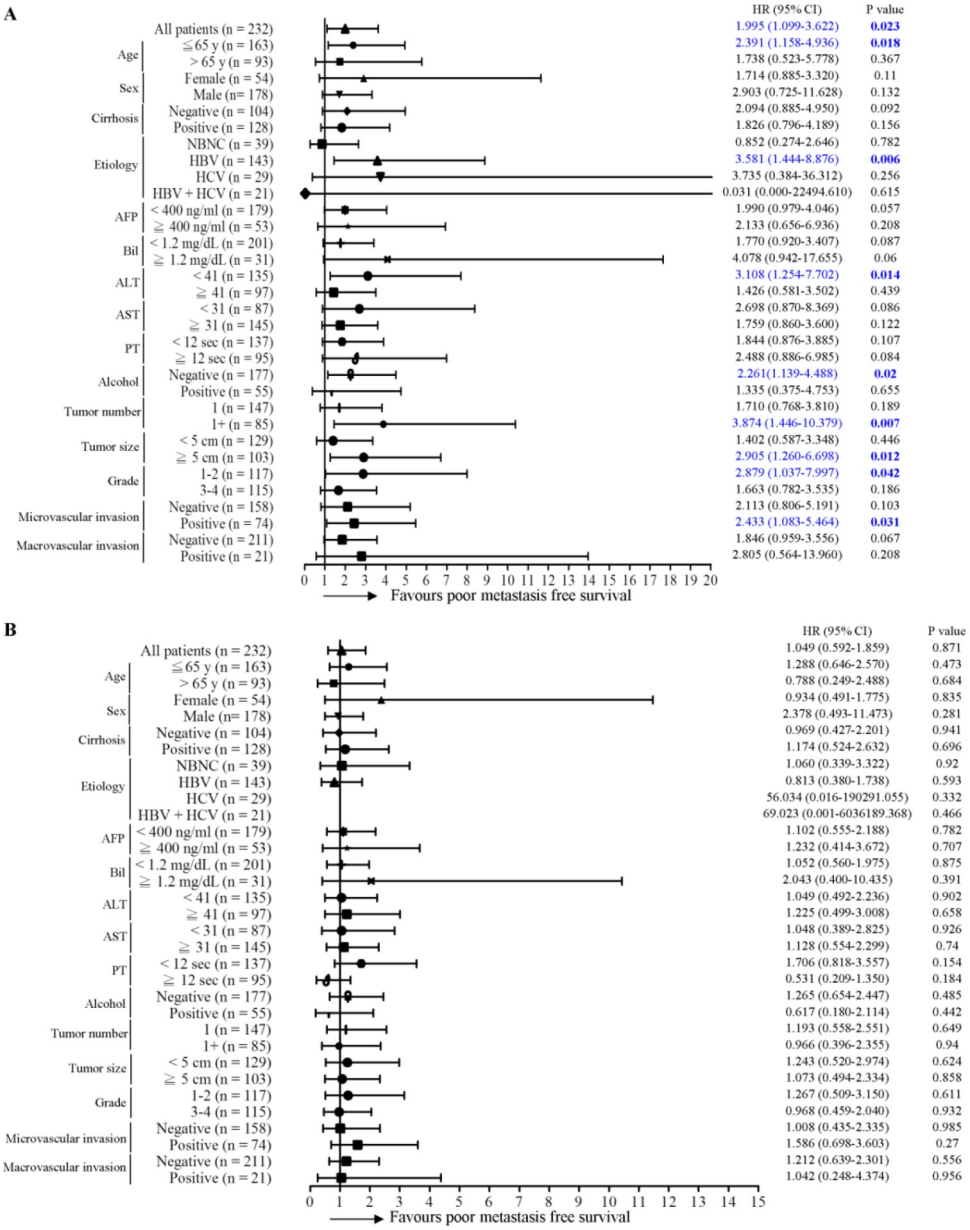
Association between γ-OHPdG expression and distant metastasis-free survival was analyzed by forest plot analysis in various clinical subgroups. The model of Cox proportional hazard method was used to analyze γ-OHPdG levels in (A) noncancerous tissues and (B) cancerous tissues in relationship to distant metastasis-free survival. Horizontal lines represent 95% confidence intervals.

**Figure 5:**
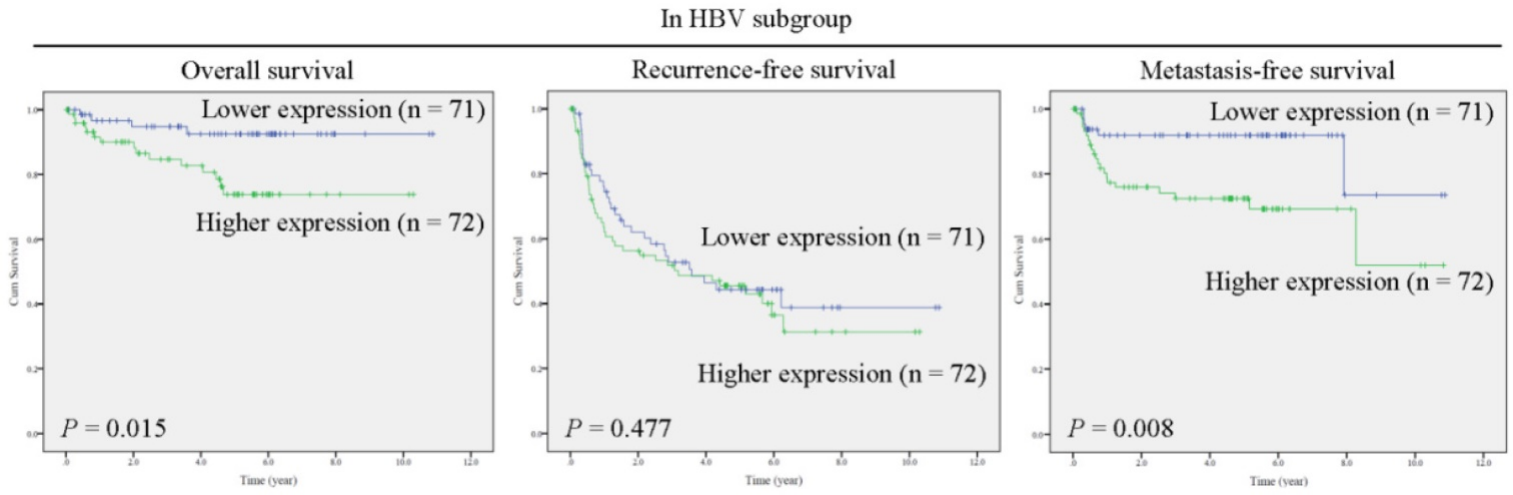
γ-OHPdG is significantly correlated with overall survival and distant metastasis-free survival in hepatitis B related HCC subgroup. Kaplan-Meier analysis of postoperative outcomes based on γ-OHPdG expression in noncancerous tissues. Median values were used as cutoffs. Survival outcomes were calculated by log-rank test.

Next, the predictive role of γ-OHPdG in several clinical parameters was determined by Cox proportional hazard method. Here, we performed a forest plot analysis. It was found that higher expression of γ-OHPdG in noncancerous tissues was significantly associated with overall survival in the following subgroups: HCC with HBV infection (HR = 3.897, 95% CI 1.293-11.743, *P* = 0.016), and tumor size < 5cm (HR = 3.732, 95% CI 1.020-13.658, *P* = 0.047) (Figure 2A). In contrast, higher expression of γ-OHPdG in cancerous tissues was significantly negatively associated with overall survival in the following subgroups: cirrhosis-negative group (HR = 0.239, 95% CI 0.184-0.914, *P* = 0.024), Bilirubin < 1.2 mg/dL (HR = 0.410, 95% CI 1.293-11.743, *P* = 0.029), ALT < 41 IU/L (HR = 0.225, 95% CI 0.075-0.673, *P* = 0.008), AST < 31 IU/L (HR = 0.072, 95% CI 0.009-0.562, *P* = 0.012), tumor number > 1 (HR = 0.272, 95% CI 0.076-0.978, *P* = 0.046) and tumor size > 5 cm (HR = 0.357, 95% CI 0.130-0.978, *P* = 0.045) (Figure 2B). For intrahepatic recurrence-free survival analysis, there was no statistically significant difference between γ-OHPdG expression, in either the noncancerous or cancerous tissues, and postoperative prognosis in any of the subgroups, except for the tumor size > 1 subgroup, wherein higher γ-OHPdG expression in the noncancerous part was associated with a shorter intrahepatic recurrence-free survival (HR = 2.326, 95% CI 1.323-4.089, *P* = 0.003) (Figure 3A and 3B). For distant metastasis-free survival analysis, higher expression of γ-OHPdG in noncancers tissues was significantly correlated with distant metastasis-free survival in the following subgroups: Age ≤65 y (HR = 2.391, 95% CI 1.158-4.936, *P* = 0.018), HBV-related HCC (HR = 3.581, 95% CI 1.444-8.876, *P* = 0.006), ALT < 41 IU/L (HR = 3.108, 95% CI 1.254-7.702, *P* = 0.014), Alcoholism-negative group (HR = 2.261, 95% CI 1.139-4.488, *P* = 0.002), tumor number > 1 (HR = 3.874, 95% CI 1.446-10.379, *P* = 0.007), tumor size ≥5 cm (HR = 2.905, 95% CI 1.260-6.698, *P* = 0.012), histology grade 1-2 (HR = 2.879, 95% CI 1.037-7.997, *P* = 0.042) and microvascular invasion positive subgroup (HR = 2.433, 95% CI 1.083-5.464, *P* = 0.031) (Figure 4A). However, there was no statistically significant difference between γ-OHPdG expression in the cancerous tissues and prognosis in each of the subgroups (Figure 4B). Considering the positive correlation between γ-OHPdG and HBV status in noncancerous tissues, we further analyzed survival outcomes in relationship to γ-OHPdG in hepatitis B related HCC subgroup. In this subgroup, Kaplan-Meier plot with log-rank analysis showed that higher expression of γ-OHPdG (median value as the cutoff) in the noncancerous tissues was significantly correlated with shorter overall survival (higher versus lower γ-OHPdG expressors, P = 0.015) and distant metastasis-free survival (higher versus lower γ-OHPdG expressors, P = 0.008), but was not associated with intrahepatic recurrence-free survival (Figure 5). Accordingly, these findings suggested that higher expression of γ-OHPdG in noncancerous tissues in some subgroups, especially in HCC patients with HBV infection, preferable contribute to patients with shorter overall survival and distant metastasis-free survival (see Figure 2 and 4, upper panels, bold P values).

Conversely, higher expressions of γ-OHPdG in cancerous tissues were associated with longer overall survival in numerous clinical subgroups (see Figure 2, lower panel, bold P values).

### Hydrogen peroxide inhibits cell viability and migration *in vitro*

**Figure 6:**
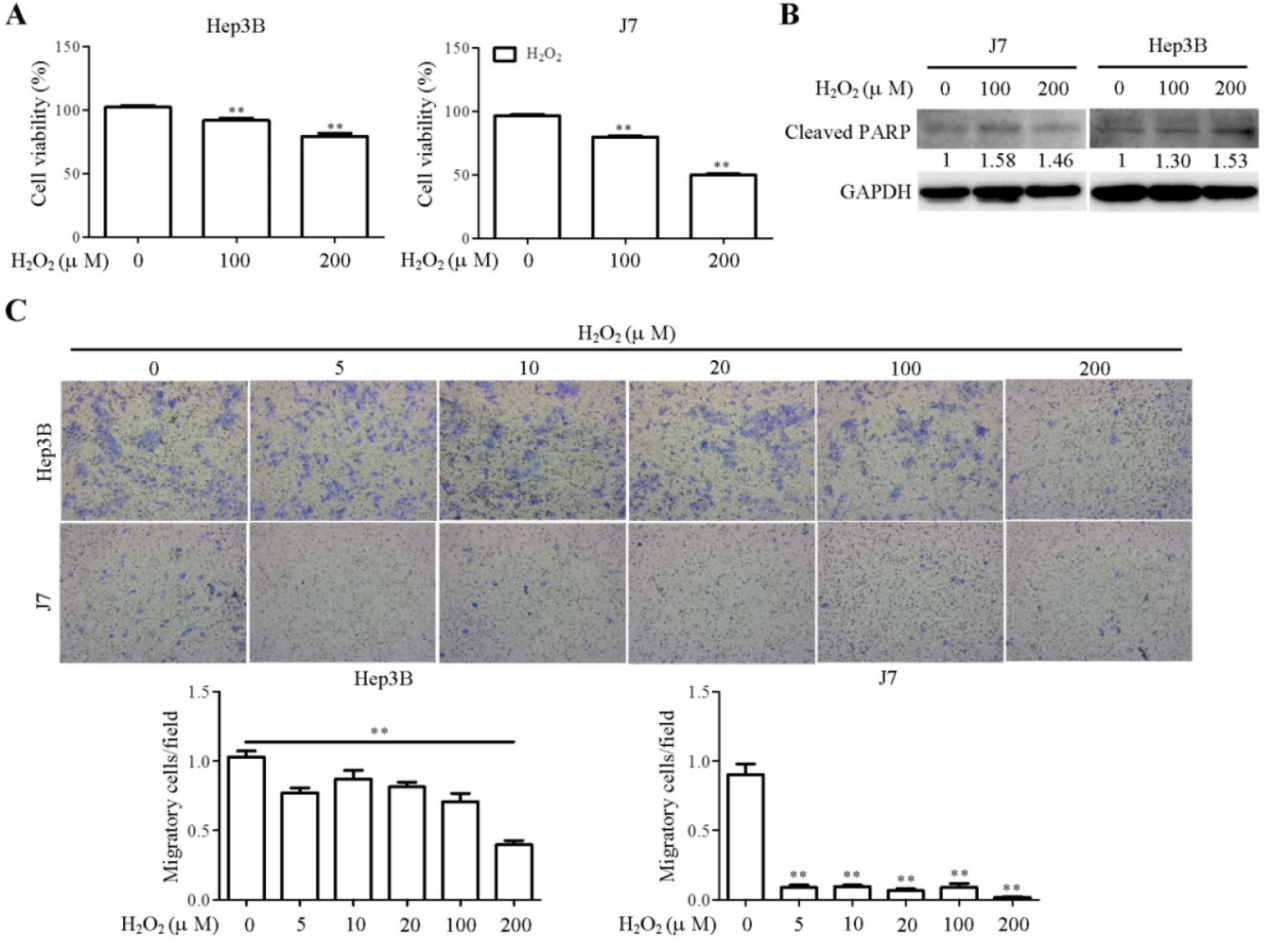
H_2_O_2_ treatment reduced cell viability and motility in hepatoma cell lines. (A) Cell viability was determined by MTT assay in Hep3B and J7 cells at 8h after H_2_O_2_ treatment. (B) The expression pattern of cleaved PARP1 in the indicated cell lines was determined by Western blot analysis, using GAPDH as a loading control. (C) Cell migration assay was measured by transwell migration assay in Hep3B and J7 cells at 18h after H_2_O_2_ treatment. The migratory cells of each group were shown and calculated as relative fold change to H_2_O_2_ 0 µM group (control group).

Hydrogen peroxide (H_2_O_2_), a ROS generator, induced cellular apoptotic pathway via oxidation of DNA, protein and lipids 17. Previous study demonstrated that H_2_O_2_ treatment triggered lipid peroxidation in rat glioma and hepatoma cell lines 18, 19. To evaluate the effect of H_2_O_2_-induced lipid peroxidation on cellular function, the cell viability and migration assays were performed in hepatoma cells upon H_2_O_2_ treatment. H_2_O_2_ treatment induced impairment of Hep3B and J7 cell viability in a dose-dependent manner (Figure 6A). During apoptosis, cleaved PARP-1 was used as a marker for apoptosis 20. We found that the cleaved PARP-1 was induced in H_2_O_2_-treated Hep3B and J7 cell lines (Figure 6B). Additionally, the results showed that H_2_O_2_ treatment significantly reduced cell migration in Hep3B and J7 cell lines (Figure 6C). Taken together, H_2_O_2_-mediated lipid peroxidation clearly suppressed cell viability and migration of hepatoma cells.

## Discussion

ROS plays important role in the development of cancer and various non-cancer diseases, including those with chronic inflammation 6. Inflammation is one of the essential biological reactions responsive to different stimuli such as infections by pathogens and contacts of toxic chemicals. Epidemiological studies indicated that some cancers were caused by chronic inflammation. In fact, chronic inflammation facilitated the processes of normal to oncogenic cell transformation. Chronic necroinflammation could induce DNA damage and genome instability, in turn, increasing the frequency of DNA mutation, leading to progression of carcinogenesis. HCC is a well-known inflammation-related cancer 21. In the current study, we provided evidences indicating that γ-OHPdG was highly expressed in HCC cancerous tissues. This result is consistent with previous findings 15. Before de novo carcinogenesis, accumulation of gene mutations caused by ROS or LPO may be involved in cell transformation. Our data indicated that γ-OHPdG levels, especially in paraneoplastic noncancerous tissues, acted as independent prognostic factor for distant metastasis-free survival. Notably, our clinical observations were externally validated in an independent cohort of good prognosis and bad prognosis in HCC patients (n = 10 per group). Expressions of γ-OHPdG levels in bad prognosis group were higher than that in good prognosis group (Data not shown). These observations are consistent with the findings shown in Figure 1. However, no statistically significant correlation was found between overall survival and γ-OHPdG expression in the cancerous HCC tissues. Accordingly, higher expression of γ-OHPdG in noncancerous tissues, and thus, higher oxidative stress, was more important in predicting postoperative distant metastasis. Furthermore, in HBV-related HCC patients, γ-OHPdG expression was associated with both distant metastasis-free and overall survivals, suggesting that oxidative stress had a more profound prognostic effect in this group of patients. Conversely, γ-OHPdG expression in the cancerous HCC tissues was associated with better overall survival in several subgroups (Figure 2), implying that oxidative stress might in fact inhibit HCC growth under some specific conditions. In this view, the oxidative stress played a cancer cell-damaging role. Our results were not completely consistent with another study indicating that high expression γ-OHPdG group was highly correlated with lower survival rates and lower intrahepatic recurrence-free survival in HCC patients 11. Ethnical and etiology differences might contribute to these inconsistencies.

Genomic instability, a hallmark of cancer, can lead to a higher than normal mutation rate. Notably, such event has been associated with poor clinical prognosis 22, 23. Mutations are caused by either genotoxic stress from cellular process or inactivation of DNA repair pathway. It has been reported that several HCC-related risk factors are able to induce formation of DNA adducts and DNA damage 24. γ-OHPdG is a DNA damage indicator and is generated by several stimulators. Previous study used Xeroderma pigmentosum group A knockout (Xpa^-/-^) mice and the diethylnitrosamine (DEN)-treated mice to investigate the association of γ-OHPdG with hepatocarcinogenesis 11. Theaphenon E, an antioxidant, treatment reduced γ-OHPdG levels in Xpa^-/-^ knockout mice and effectively repressed HCC formation in DEN-treated mice. This result was in agreement with our observation that γ-OHPdG in the noncancerous parts was correlated with postoperative distant metastasis (and overall survival in HBV-related HCC patients). Another study demonstrated that mitochondrial functions, including mitochondrial DNA damage and respiratory chain complex IV inhibition, were directly associated with LPO in carbon tetrachloride-treated mice model 25. NER is the main DNA repair system for repairing these DNA adducts 26, 27. Previous findings demonstrated that HBx suppressed NER pathway through inhibition of xeroderma pigmentosum type B (XPB) and type D (XPD) helicase in patients with HBV, resulting in inefficiently removing these DNA adducts 28, 29. These findings suggest that HBV infections not only cause DNA mutation but also reduce DNA repair pathway, thus leading to a synergistic effect on HCC progression. Consistently, in our forest plot analysis, we found that γ-OHPdG expression in noncancerous tissues was stronger correlated with overall survival and distant metastasis-free survival in the HBV-related subgroups but not the other etiology subgroups (NBNC, HCV and HBV + HCV).

In fact, the biological functions of ROS in cancer development have been a controversial issue. We found that H_2_O_2_ treatment in hepatoma cells suppressed cell viability and motility, indicating that H_2_O_2_-mediated ROS/lipid peroxidation functioned as a negative regulator to repress tumor formation. Clinically, higher γ-OHPdG expression in cancerous liver tissues was correlated with a better overall survival. Conversely, higher expression of γ-OHPdG in the noncancerous tissues was correlated with shorter overall survival in hepatitis B related HCC. Presumably, ROS-induced γ-OHPdG production could trigger noncancerous cells turnover/regeneration, which thus contributed to enhanced cell transformation leading to increased distant metastasis and shorter overall survival. In conclusion, assessment of γ-OHPdG expression in paraneoplastic noncancerous and cancerous liver tissues is helpful in predicting the postoperative outcomes of HCC, especially for HBV-related HCC patients.

## Figures and Tables

**Table 1:** Basic clinicopathological factors of patients included

Variables	Patient numbers
**Age (years)**	
<65	153 (65.9%)
≥65	79 (34.1%)
**Gender**	
Male	178 (76.7%)
Female	54 (23.3%)
**Cirrhosis**	
No	104 (44.8)
Yes	128 (55.2)
**AFP**	
< 400 ng/ml	179 (77.1%)
≥ 400 ng/ml	53 (22.9%)
**Viral status**	
NBNC	39 (16.8%)
HBV	143 (61.6%)
HCV	29 (12.5%)
HBV+HCV	21 (9.1%)
**Bilirubin**	
< 1.2 mg/dL	201 (86.6%)
≥ 1.2 mg/dL	31 (13.4%)
**ALT**	
< 41 IU/L	135 (58.1%)
≥ 41 IU/L	97 (41.9%)
**AST**	
< 31 IU/L	87 (37.5%)
≥ 31 IU/L	145 (62.5%)
**Prothrombin time**	
< 12 sec	137 (59.1%)
≥ 12 sec	95 (40.9%)
**Alcoholism**	
Negative	177 (76.3%)
Positive	55 (23.7%)
**Tumor number**	
1	147 (63.4%)
> 1	85 (36.6%)
**Tumor size**	
< 5 cm	129 (55.6%)
≥ 5 cm	103 (44.4%)
**Grade**	
1-2	117 (50.4%)
3-4	115 (49.6)
**Microvascular invasion**	
Negative	158 (68.1%)
Positive	74 (31.9%)
**Macrovascular invasion**	
Negative	211 (90.9%)
Positive	21 (9.1%)

**Table 2:** Univariate and multivariate analyses of distant metastasis-free survival in HCC patients (n = 232) by Cox regression analysis

Variables	Univariate Analysis		Multivariate analysis	
	HR (95% CI)	*P*	HR (95% CI)	*P*
Gender	1.293 (0.624-2.676)	0.489		
Age	1.332 (0.708-2.505)	0.373		
Cirrhosis	0.784 (0.442-1.390)	0.405		
HBV	0.696 (0.383-1.265)	0.234		
HCV	0.747 (0.348-1.604)	0.455		
AFP	1.583 (0.847-2.960)	0.150		
Bilirubin	1.273 (0.592-2.739)	0.537		
ALT	0.912 (0.511-1.628)	0.756		
AST	1.123 (0.613-2.054)	0.708		
Prothrombin time	0.827 (0.456-1.500)	0.531		
Alcoholism	1.006 (0.512-1.977)	0.986		
Tumor number	1.295 (1.013-1.655)	0.039	1.178 (0.932-1.489)	0.171
Tumor size	1.776 (0.998-3.162)	0.051		
Grade	1.350 (0.896-2.034)	0.151		
Microvascular invasion	2.837 (1.616-5.106)	0.000	2.711 (1.616-5.106)	0.001
Macrovascular invasion	2.716 (1.307-5.643)	0.007	3.360 (1.106-10.209)	0.033
γ-OHPdG (noncancerous tissue)	1.995 (1.099-3.622)	0.023	2.758 (1.104-6.889)	0.030
γ-OHPdG (tumor)	1.049 (0.592-1.859)	0.871		

Abbreviations: HR: hazard ratio; CI: confidence interval.

## Abbreviations

AFP: alpha-fetoprotein
ALT: alanine transaminase
AST: aspartate transaminase
CI: confidence interval
DEN: diethylnitrosamine
FFPE: Formalin-fixed paraffin embedded
HBV: hepatitis B virus
HCV: hepatitis C virus
HCC: hepatocellular carcinoma
HR: hazard ratios
IHC: immunohistochemistry
LPO: lipid peroxidation
Nrf1: nuclear respiratory factor-1
NER: Nucleotide excision repair
OS: overall survival
γ-OHPdG: γ-hydroxy-1, N2-propanodeoxyguanosine
RFS: recurrence-free survival
ROS: reactive oxygen species
